# Care-seeking behaviour and socio-economic burden associated with uncomplicated malaria in the Democratic Republic of Congo

**DOI:** 10.1186/s12936-021-03789-w

**Published:** 2021-06-09

**Authors:** Nadine Kalenda Kayiba, Doudou Malekita Yobi, Brecht Devleesschauwer, Dieudonné Makaba Mvumbi, Pius Zakayi Kabututu, Joris Losimba Likwela, Lydie Azama Kalindula, Patrick DeMol, Marie-Pierre Hayette, Georges Lelo Mvumbi, Paul Dikassa Lusamba, Philippe Beutels, Angel Rosas-Aguirre, Niko Speybroeck

**Affiliations:** 1 Research Institute of Health and Society (IRSS), Université Catholique de Louvain, Brussels, Belgium; 2 School of Public Health, Faculty of Medicine, University of Kinshasa, Kinshasa, Democratic Republic of Congo; 3 School of Public Health, Faculty of Medicine, University of Mbujimayi, Mbujimayi, Democratic Republic of the Congo; 4 Department of Basic Sciences, Faculty of Medicine, University of Kinshasa, Kinshasa, Democratic Republic of the Congo; 5 Department of Epidemiology and Public Health, Sciensano, Brussels, Belgium; 6 Department of Quality of Laboratories, Sciensano, Brussels, Belgium; 7 Department of Veterinary Public Health and Food Safety, Ghent University, Ghent, Belgium; 8 National Malaria Control Programme, Kinshasa, Democratic Republic of the Congo; 9 Laboratory of Clinical Microbiology, Center for Interdisciplinary Research on Medicines (CIRM), University of Liège, Liège, Belgium; 10 Centre for Health Economics Research and Modelling Infectious Diseases, University of Antwerp, Antwerp, Belgium

**Keywords:** Malaria, Cost-of-illness, Health-related quality of life, National Malaria Control Programme, Democratic Republic of Congo

## Abstract

**Background:**

This study aimed to estimate the socio-economic costs of uncomplicated malaria and to explore health care-seeking behaviours that are likely to influence these costs in the Democratic Republic of Congo (DRC), a country ranked worldwide as the second most affected by malaria.

**Methods:**

In 2017, a cross-sectional survey included patients with uncomplicated malaria in 64 healthcare facilities from 10 sentinel sites of the National Malaria Control Programme (NMCP) in the DRC. A standard questionnaire was used to assess health care-seeking behaviours of patients. Health-related quality of life (HRQL) and disutility weights (DW) of illness were evaluated by using the EuroQol Group’s descriptive system (EQ-5D-3L) and its visual analogue scale (EQ VAS). Malaria costs were estimated from a patient’s perspective. Probabilistic sensitivity analyses (PSA) evaluated the uncertainty around the cost estimates. Generalized regression models were fitted to assess the effect of potential predictive factors on the time lost and the DW during illness.

**Results:**

In total, 1080 patients (age: 13.1 ± 14 years; M/F ratio: 1.1) were included. The average total costs amounted to US$ 36.3 [95% CI 35.5–37.2] per malaria episode, including US$ 16.7 [95% CI 16.3–17.1] as direct costs and US$ 19.6 [95% CI 18.9–20.3] indirect costs. During care seeking, economically active patients and their relatives lost respectively 3.3 ± 1.8 and 3.4 ± 2.1 working days. This time loss occurred mostly at the pre-hospital stage and was the parameter associated the most with the uncertainty around malaria cost estimates. Patients self-rated an average 0.36 ± 0.2 DW and an average 0.62 ± 0.3 EQ-5D index score per episode. A lack of health insurance coverage (896 out of 1080; 82.9%) incurred substantially higher costs, lower quality of life, and heavier DW while leading to longer time lost during illness. Residing in rural areas incurred a disproportionally higher socioeconomic burden of uncomplicated malaria with longer time lost due to illness and limited access to health insurance mechanisms.

**Conclusion:**

Uncomplicated malaria is associated with high economic costs of care in the DRC. Efforts to reduce the cost-of-illness should target time lost at the pre-hospital stage and social disparities in the population, while reinforcing measures for malaria control in the country.

**Supplementary Information:**

The online version contains supplementary material available at 10.1186/s12936-021-03789-w.

## Background

Malaria remains a major public health concern with its highest burden occurring in Africa, where more than 200 million cases are reported yearly [1]. Beside its high morbidity and mortality in the continent, malaria diverts substantial financial resources from individuals and countries towards prevention and treatment efforts [2, 3]. Consequently, the malaria burden weighs heavily on households, the healthcare system, and economic growth and development [3–5]. While affecting the health and wealth of nations, malaria is both a disease of poverty and a cause of poverty in endemic countries [5]. This is particularly the case in the Democratic Republic of Congo (DRC), which accounts for a tenth of the burden of malaria in Africa with over 15 million cases and 25 thousand deaths recorded every year [1, 6, 7]. Nearly all its population lives in high transmission zones where malaria is caused mainly by *Plasmodium falciparum*, the deadliest species linked to severe complications especially in children under five [6, 7].

The DRC is the largest and most populated country in Middle Africa, with an estimated 89 million inhabitants living mostly in rural areas (61.6%) [8]. With a Gross Domestic Product (GDP) of US$ 514/year/inhabitant in 2017, the DRC is among the countries with the lowest income worldwide (https://tradingeconomics.com/congo/indicators; accessed on 12 March 2020). Total expenditure on health in the country is low (e.g., only US$ 13 per capita per year in 2012) with households being the largest contributors, closely followed by multilateral aid, while the central government contributes only 11% [9]. The health system in the country is organized in a pyramidal structure with three levels: the National Ministry of Public Health (i.e., central level), provincial health departments (i.e., intermediate level), and health districts (i.e., peripheral level) [9, 10]. To specifically reduce the burden of malaria in the country, a National Malaria Control Programme (NMCP) was implemented within the national health system in the 2000s and relies mainly on external funding for its activities [7]. The most up-to-date anti-malarial policies that are outlined in the National Malaria Control Strategic Plan (NSP) 2016–2020 recommend free universal access to malaria prevention, diagnosis, and treatment services in different health facilities [11, 12]. All health districts and respective provincial health departments currently integrate the national anti-malarial policy and thus connect to the NMCP. Subsequent to the deployment of national policies, significant progress in terms of the country coverage by malaria control services and reduced cases was noted in 2010–2015 [7].

However, despite the NMCP’s efforts, patients with malaria still face several barriers to accessing health services, including economic costs resulting from lost productivity due to the impact of illness or incurred to afford care during the infection episode [8, 13]. With less than 10% of the population covered by formal health insurance programmes, the national health system relies heavily on households’ direct contributions even for diseases, such as malaria that receive substantial multilateral aid [9, 10]. Patients can thus be deterred from utilizing malaria health care by high care costs that may drive the use of less effective care or practices [10, 14]. This situation leads to equity issues between regions that impact the quality of life of populations [9, 15]. Ascertaining costs of malaria care and associated factors can provide essential information for soliciting appropriate funding for its control, from both government and non-government organizations, to reduce any out-of-pocket expenditure in the population. Moreover, identifying key factors that affect health care costs would be critical for decision-making, priority setting, and resource allocation [9, 16]. The country’s income, geographic location, and health care strategies have thus been widely described as factors that influence the economic costs of malaria at the population scale [16]. At the level of individuals, health care-seeking behaviours (e.g., self-medication, delayed behaviours) linked to personal beliefs and health awareness have been reported to influence the cost-of-illness [17–19]. The clinical form of malaria—i.e., severe or uncomplicated malaria—may also substantially affect the disease burden in endemic regions. In fact, unlike the severe form of malaria that is limited to specific groups at high risk (e.g., children under five years, pregnant women, immunocompromised patients), uncomplicated malaria exhibits an excellent prognosis within 48 to 72 h after early diagnosis and prompt initiation of an effective treatment [20, 21]. However, this clinical form of malaria may account for the highest burden due to its high incidence and the occurrence of multiple intermittent episodes in the same individuals over the time [21]. However, in DRC, only limited studies have been conducted to assess the costs of malaria, focusing mainly on severe malaria and on malaria episodes at small scales (e.g., single hospital or single city) [14, 15, 22, 23]. Therefore, this study aimed to estimate the socioeconomic burden of uncomplicated malaria across the DRC while exploring health care-seeking behaviours that are likely to influence the cost-of-illness.

## Methods

### Study setting

In the DRC, the peripheral level of the health system comprises 516 health districts, where a district team manages a network of health centres and a district hospital typically covering a population of 100,000 to 200,000 [9, 10]. At the intermediate level, a provincial health department is responsible for technical and logistical support of health services in each of the country’s 26 provinces. A coordination or a direction integrated in the National Ministry of Public Health has a normative role and constitutes the central level of the health system [9, 10]. Health districts are classified as rural or urban based on their geographical location. Compared to urban districts, rural areas are characterized by more poverty, private for-profit health care service delivery points, less qualified health personnel, inefficient workforce deployment, difficulties in transportation, and insufficient social services, but also less promiscuity [9]. Healthcare facilities are central to the provision of malaria treatment and hence critical to the success of its management in the country [11]. The recommended drugs for uncomplicated malaria are a combination of artesunate + amodiaquine (ASAQ) or that of artemether + lumefantrine (AL) [12]. The NMCP manages malaria activities in each health district and routinely monitors the efficacy of malaria control strategies through a network of sentinel sites set in 26 selected health districts [10, 12]. This study was carried out in ten of these NMCP sentinel sites to capture the cost-of-illness in both urban and rural conditions during uncomplicated malaria.

### Study participants

This study targeted patients with uncomplicated malaria who sought care and received treatment at health facilities in selected sites. An uncomplicated malaria case was defined as any symptomatic patient with a diagnosis of malaria confirmed by microscopy or rapid diagnostic test, whose clinical status did not require hospitalization. Patients with known co-morbidities (i.e., other diagnosed chronic or acute illness) or documented pregnancy were excluded from the study to allow, as far as possible, a specific evaluation of the impact of malaria on patients’ quality of life.

### Study design and data collection

**Fig. 1:**
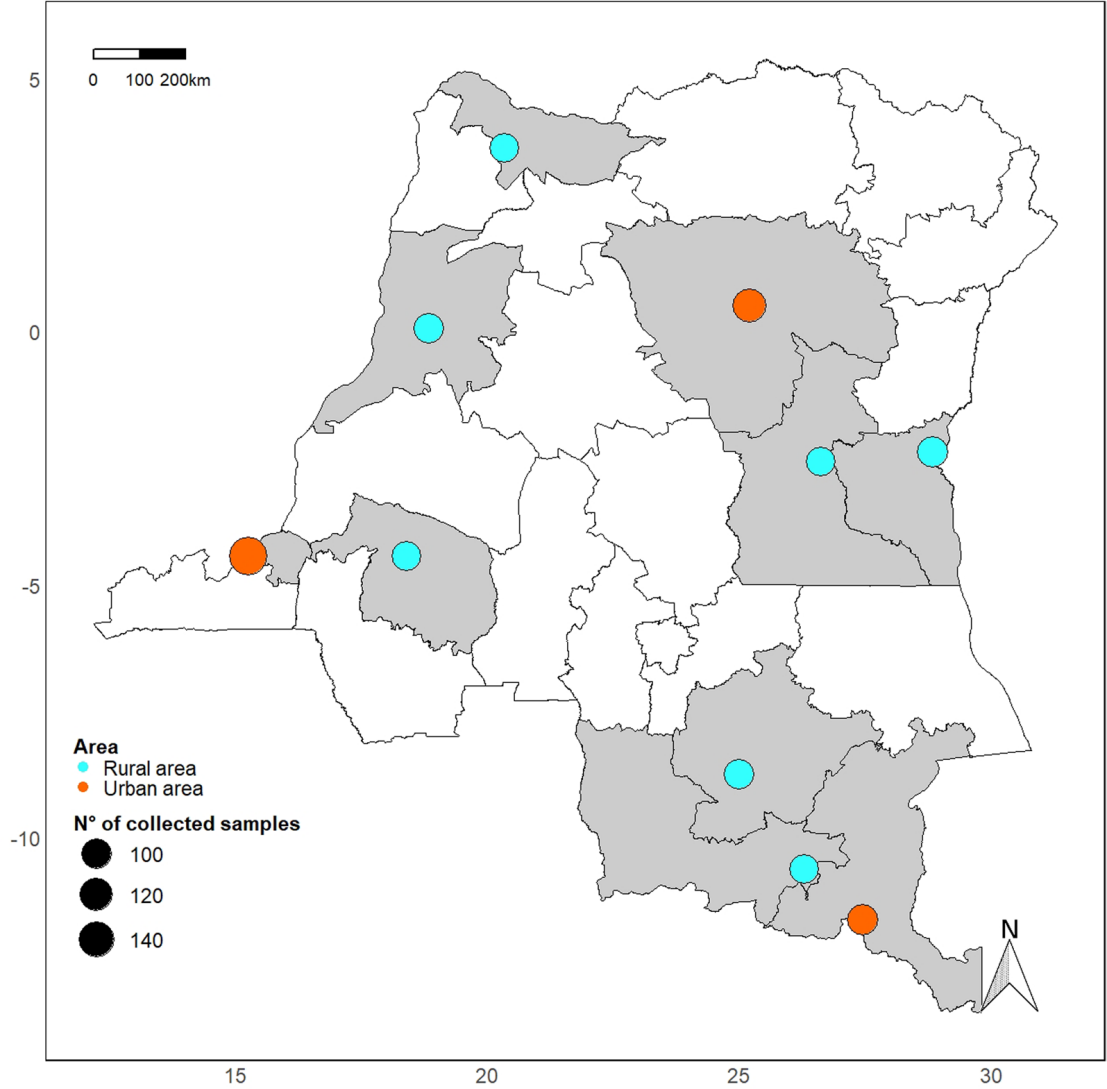
Geographic map of sampling sites in Democratic Republic of Congo. This map displays each site where the survey took place. Unshaded regions represent provinces from which no sample was collected during this study while regions shaded in gray represent surveyed provinces. Circles represent the sample size of patients included in each NMCP sentinel site selected for the survey, with orange circles being located in urban areas and aqua circles in rural areas

A cross-sectional survey was conducted from January to December 2017 in 10 of the 26 NMCP sentinel sites, selected based on their accessibility and functionality for malaria surveillance activities. Three of these sites were located in urban areas (Kinshasa, Lubumbashi, and Kisangani) and seven in rural areas (Vanga, Bolenge, Karawa, Kalima, Fungurume, Kamina, and Katana) (Fig. 1). In total 64 healthcare facilities were involved in the data collection. At each collection site, the field research team consisted of the head nurse in charge of malaria activities for each corresponding health district, a general practitioner, a nurse, and a laboratory technician recruited from a local healthcare facility. All teams were initially trained to conduct interviews using study questionnaires [See Additional file 1]. Beforehand, the questionnaires were pre-tested and translated into national languages spoken in the different sites (i.e., French, Lingala, Kikongo, and Kiswahili) and back-translated to English. The data collected included socio-economic characteristics, general knowledge on malaria, malaria-related attitudes and practices at the household level, information about previous and current malaria episodes with details about the trajectory followed during the care-seeking, as well as expenditures and time lost due to the current malaria episode. In addition, the health-related quality of life (HRQL) was assessed for each patient on arrival at the healthcare facility using the EuroQol Group’s descriptive system (EQ-5D-3L) and its visual analogue scale (EQ VAS) [24, 25]. An associated investigator reviewed the collected data daily and supervised research teams on the field. Data were double entered, cross-checked, and corrected in an Excel spreadsheet.

### Estimation of economic costs

Total economic costs of uncomplicated malaria episodes in DRC were estimated for the year 2017 in two steps. First, case-based parameters (i.e., cost parameters or unit costs) were obtained through the field survey following a bottom-up approach measuring health cost inputs from the patient’s perspective. A “bottom-up” approach estimates total costs for specific health care services by relying on expense inputs recorded at the level of individuals, rather than aggregated data at population level that would be required for a “top-down” method [26]. Costs from the “patient’s perspective” are defined as expenses estimated according to the patients, their experience of health care services, and self-perceived impact of the health condition on their life, as well as their expectations of the consultation or the caregivers, and their priorities regarding the outcomes of treatment [27]. Two categories of input were used for estimating total average costs, including direct and indirect costs of care for uncomplicated malaria [26, 27]. In the second step, total costs for the country were estimated by extrapolating average costs to all malaria episodes recorded across the country by the NMCP in 2017. Unit costs initially obtained in Congolese Francs (CDF) were converted to United States Dollars (US$) using the average exchange rate in 2017 (one US$ = CDF 1648).

### Direct costs

Direct costs were defined as out-of-pocket health expenditures paid by a patient and his/her relatives for medical products and services (i.e., direct medical costs) used to detect and treat the malaria episode, as well as costs for non-medical services (i.e., direct non-medical costs) that are the result of illness [26, 27]. Resources for these out-of-pocket expenditures were sought at both the pre-hospital stage (i.e., outside the circuit of formal health establishments and before undertaking a medical consultation) and the hospital stage (i.e., after the medical consultation). Costs incurred at the hospital stage included fees for the medical consultation, laboratory analyses (diagnosis by RDTs and other biological tests), treatment (i.e., anti-malarial drugs, antipyretics, analgesics, vitamins, and care administration), and follow-up. Follow-up costs included costs of a second medical consultation and a microscopic examination scheduled three days after the start of anti-malarial treatment according to national malaria treatment guidelines. Direct non-medical costs investigated in this study included transportation costs for a patient and his/her relatives, and other costs resulting from the illness (including telephone calls, food consumed during the care-seeking process).

### Indirect costs

Indirect costs of malaria were defined as potential losses of productivity or work income that economically active (EA) individuals incurred due to illness [26]. Therefore, cost estimation multiplied the reported time lost from work in days (cost parameter expressed in person-days) by the amount of money lost in 1 day for not working (person-day unit cost). Person-day unit costs (US$ 5.0) for patients, relatives, and eventual substitutes in the workplace were calculated using the monthly minimum wage of 2017 as reference, by dividing it by the number of working days per month (http://www.fec-rdc.com/index.php/nos-publications/category; accessed on 15 December 2019). EA individuals were defined as those who were ≥ 15 years old (i.e., the economically active sub-population) [28]. Time losses included in the cost estimation were obtained by interrogating the waiting time while undertaking health care, days off from daily activities (school or work) due to illness (in the patient’s case) or due to the need to support a patient’s care (in the case of the patient’s relatives).

### Average cost per episode and total costs

The direct and indirect costs obtained from the patient’s perspective yielded the average total cost per episode. Costs of all malaria episodes per patient during one year were achieved by multiplying the average total costs by the number of reported putative malaria episodes in a year. The total costs for the country in 2017 were obtained by multiplying average total costs by the number of malaria episodes recorded in the database of the National Health Information System (SNIS) of the NMCP for that year.

### Evaluation of the quality of life and disutility weight during malaria episodes

The health-related quality of life (HRQL) during uncomplicated malaria was evaluated using a EQ-5D toolkit developed by the EuroQol Group [24, 25]. This toolkit comprises a three-level version of the EQ-5D system (EQ-5D-3L) and a visual analogue scale (EQ VAS) that allows health outcomes to be measured based on the patient’s own judgment [29]. The EQ-5D-3L is a descriptive system that classifies health status into three levels (i.e., no problems, some problems, and extreme problems) across each of its five dimensions: mobility, self-care, usual activities, pain/discomfort, and anxiety/depression. EQ-5D-3L health states were then converted into index “utility” scores using the algorithm established based on societal preference values defined for the Zimbabwean population [30]. Currently, in Africa the Zimbabwean algorithm is the only one that is available for converting the EQ-5D-3L questionnaire into a single EQ-5D index score. The formula DW = 1–(VAS score/100) was used to estimate the disutility weight (DW) on the basis of the patient’s self-rated EQ VAS visual scale with endpoints from 100 “best imaginable health state” to 0 “worst imaginable health state” [31].

### Data analysis

Data analysis was carried out using R software version 3.5.3 (The R Development Core Team, R Foundation for Statistical Computing, Vienna, Austria, 2019). Absolute and relative frequencies were used to summarize qualitative variables. Averages with corresponding standard deviations (SD) were used to describe quantitative characteristics of patients, while means with standard errors (SE) described the deviation inferred on the population. When appropriate, qualitative variables were compared between groups using the Chi-square test or the Fisher exact test. In contrast, quantitative variables were compared using parametric (e.g., ANOVA test or *t*-test) or non-parametric tests (Kruskal–Wallis rank sum test or Wilcoxon rank sum test with continuity correction), the normality and homogeneity of variances in distributions being tested with the Shapiro–Wilk test and the Levene test. Moreover, uni- and multivariable generalized linear models (GLMs) were fitted to assess the effect of potential predictive factors on the time lost and the DW during uncomplicated malaria. A Gamma distribution and a Beta distribution with a log-link function were respectively assumed to model the time lost and the DW. Whenever a multivariable model was considered, the final model was selected based on Akaike's “An Information Criterion” (AIC) values calculated for GLMs in a stepwise algorithm using both backward and forward directions. A significance level of p < 0.05 was considered for all statistical tests.

Multi-way probabilistic sensitivity analysis (PSA) assessed the confidence of estimated average costs per uncomplicated malaria episode in DRC (total costs for the country in 2017 divided by the total number of episodes). This took into account the uncertainty that surrounded the most relevant base case parameters (i.e., point estimates for cost parameters and unit costs obtained in the survey) by defining probability distributions for them as described elsewhere [32]. The beta distribution was chosen for the probability of being economically active either among malaria patients or relatives; while the gamma distribution was used for unit costs (e.g., unit costs related to malaria diagnosis and treatment) and the time lost from work due to a malaria episode. Then, 10,000 Monte Carlo simulations were conducted to propagate uncertainty by sampling values from all the probability distributions jointly and calculating the corresponding values for the cost per malaria episode. Finally, upper and lower 95% uncertainty limits (2.5 and 97.5%) for the cost per malaria episode were obtained from these simulations and reported using tornado diagrams.

## Results

### Socio-economic characteristics of patients with uncomplicated malaria in the DRC

Overall, 1080 malaria patients (age: 13.1 ± 14.0 years; ratio M:F = 1.1), mostly living in rural areas (n = 688; 63.7%), were enrolled in 64 healthcare facilities, from the 10 selected NMCP sentinel sites (Table 1). There was no difference in gender (p = 0.509) and education level (p = 0.163) between patients from rural and urban areas. Patients from rural areas were older (average age: 14.9 ± 14.9; p < 0.001), less covered by a health care insurance (4.7%; p < 0.001), more frequently among the most economically disadvantaged patients (163 of 219 patients of the poorest quintile; p < 0.001), and more likely to be treated in a private for-profit health facility (62.9%; p < 0.001) than those from urban areas. The proportion of malaria patients holding health insurance policies was significantly lower in female subjects than males (14.3% and 19.9%; p = 0.017), in adult than young patients (12.5% and 19.2%; p = 0.008), and in those with the lowest education level than the most educated ones (0.0% and > 13%; p < 0.02). In addition, substantially fewer health insurance holders were observed among patients from rural areas than those from urban settings (4.7% and 38.8%; p < 0.001), among those from the most economically disadvantaged category than others (only 0.2% and > 12%; p < 0.001), and in those seeking care in private for-profit healthcare facilities than in conventional ones (11.6% and 25.9%; p < 0.001) [See Additional file 2].

**Table 1:** Baseline socio-economical characteristics of patients with uncomplicated malaria in the DRC

Characteristics	Rural area		Urban area		Total		p-value
	n = 688		n = 392		n = 1080		
	n	%	n	%	n	%	
Gender							0.509
Female	358	52	195	49.7	553	51.2	
Male	330	48	197	50.3	527	48.8	
Education level (n = 257; age ≥ 18 years)							0.163
No education	31	14.6	7	9.3	38	13.2	
Primary level	36	17	8	10.7	44	15.3	
High school level	117	55.2	44	58.7	161	56.1	
College or University level	28	13.2	16	21.3	44	15.3	
Age group (years)^a^							< 0.001
[0–5]	244	35.5	146	37.2	390	36.1	
[5–15]	187	27.2	152	38.8	339	31.4	
≥ 15	257	37.4	94	24	351	32.5	
Socio-economic index quintile							< 0.001
Quintile 1 "most economically disadvantaged"	163	23.7	56	14.3	219	20.3	
Quintile 2 "very economically disadvantaged"	94	13.7	125	31.9	219	20.3	
Quintile 3 "economically disadvantaged"	151	21.9	65	16.6	216	20	
Quintile 4 "less economically disadvantaged"	151	21.9	68	17.3	219	20.3	
Quintile 5 "least economically disadvantaged"	129	18.8	78	19.9	207	19.2	
Health insurance status							< 0.001
Yes	32	4.7	152	38.8	184	17.1	
No	656	95.3	240	61.2	896	82.9	
Type of healthcare facility							< 0.001
Conventional	255	37.1	192	49.0	447	41.4	
Private for-profit	433	62.9	200	51.0	633	58.6	

^a^Average ages of patients in rural and urban areas were 14.9 ± 14.9 and 11.0 ± 12.1 years old, respectively (p < 0.001)

### Malaria knowledge, attitude, and practices of malaria patients in the DRC

Although most respondents knew the main mechanism of malaria transmission (mosquito bite: 83.1%), there were people who were still unaware of this mechanism (7.1%) or were still evoking inappropriate mechanisms (11.5%), including: non-potable drinking water, witchcraft, lack of hygiene and sanitation, contaminated food, and bites from various insects. Fever (84.9%), headache (0.3%), chills (33.8%), and generalized pain (22.3%) were the most common malaria symptoms identified by respondents. Mosquito bed nets were evoked as the most effective method for malaria prevention (84.4%). However, 7.3% of respondents were still convinced that there is no effective prevention method, while 1.1% of them were still unaware of any existing prevention approach. Overall, respondents from rural areas had better knowledge of malaria transmission mechanisms and prevention methods (p < 0.001) [See Additional file 3]. Mosquito bed nets were available in most of respondents’ households (about 90%) and had been acquired mostly during the past three years (in around 95%) [See Additional file 4]. However, only 76.7% of patients (81.7% among children under 5 years old) reported having slept under a bed net the night before the survey.

### Health care-seeking behaviour during malaria episodes in the DRC

More than half of the patients (57.8%) had at least one malaria episode in the past year besides the episode diagnosed at the time of the survey. The average number of malaria episodes per participant reported by patients in a year was 2.7 ± 1.6. The most recent malaria episode reported by participants dated back 3.7 ± 2.7 months before the survey, but 19.0% of them were not tested for malaria and 6.7% did not take any anti-malarial drug. During the episode diagnosed at the time of the survey, patients lost 3.3 ± 1.8 days, which represented about half of the total duration of illness (~ six days) and occurred mainly at the pre-hospital stage. Upon arrival in hospitals, the majority of patients (68.7%) had already attempted to treat their illness. Treatment at pre-hospital stage mainly came from the informal sector (~ 72% of cases), including an anti-malarial drug in nearly one out of four cases (26.3%) [See Additional file 5].

### Social burden and quality of life during uncomplicated malaria illness in the DRC

More than half of the patients (> 50%) with uncomplicated malaria episodes reported moderate to extreme problems in different dimensions of HRQL defined by the EQ-5D-3L questionnaire. These problems were more frequent in urban than in rural areas during malaria episodes, especially in dimensions related to self-care, usual activity, and anxiety or depression (p < 0.001) [See Additional file 6]. Malaria patients reported an average 0.36 ± 0.2 DW on the EQ VAS and an average 0.62 ± 0.3 EQ-5D index score. Generalized linear models predicted a significantly increased DW in patients with no health insurance coverage (p = 0.005) while average EQ-5D index scores were significantly lower in young patients (p < 0.001), in those not covered by health insurance (p < 0.001), and those residing in rural areas (p < 0.009) (Tables 2,  3).

**Table 2:** Comparison of EQ-5D index score in different categories of patients with uncomplicated malaria in the DRC

Variable	n	Average (SD)	p-value
All patients	1080	0.62 (0.26)	–
Age group			
Adult patients	351	0.65 (0.25)	< 0.001
Young patients	729	0.61 (0.27)	
Health insurance coverage			
Yes	184	0.71 (0.16)	< 0.001
No	896	0.61 (0.27)	
Type of healthcare facility			
Conventional	447	0.6 (0.3)	0.144
Private for-profit	633	0.65 (0.23)	
Residence			
Rural area	688	0.62 (0.23)	0.009
Urban area	392	0.63 (0.27)	
Socioeconomic index quintile			
Quintile 1 "most economically disadvantaged"	219	0.62 (0.26)	0.222
Quintile 2 "very economically disadvantaged"	219	0.62 (0.25)	
Quintile 3 "economically disadvantaged"	216	0.61 (0.28)	
Quintile 4 "less economically disadvantaged"	219	0.62 (0.25)	
Quintile 5 "least economically disadvantaged"	207	0.65 (0.26)	
Gender			
Female	553	0.63 (0.27)	0.451
Male	527	0.62 (0.25)	

SD, standard deviation;

**Table 3:** Beta generalized linear regression model for the disutility weight (DW) during uncomplicated malaria in the DRC

Patients	n	Average (SD)	MR [95% CI]	p-value
Gender				
Male	527	0.36 (0.20)	Ref	–
Female	553	0.37 (0.20)	1.0 [0.9; 1.1]	0.654
Age (years)	1080	0.36 (0.20)	0.9 [0.9; 1.0]	0.211
Residence				
Rural area	688	0.36 (0.20)	Ref	–
Urban area	392	0.37 (0.21)	1.1 [0.9; 1.1]	0.126
Health insurance coverage				
Yes	184	0.32 (0.18)	Ref	–
No	896	0.37 (0.20)	1.2 [1.0; 1.3]	0.005
Socioeconomic index quintile				
Quintile 1 "most economically disadvantaged"	219	0.35 (0.19)	Ref	–
Quintile 2 "very economically disadvantaged"	219	0.36 (0.20)	1.1 [0.9; 1.2]	0.389
Quintile 3 "economically disadvantaged"	216	0.37 (0.20)	1.1 [0.9; 1.2]	0.118
Quintile 4 "less economically disadvantaged"	219	0.38 (0.21)	1.1 [1.0; 1.3]	0.019
Quintile 5 "least economically disadvantaged"	207	0.36 (0.20)	1.0 [0.9; 1.2]	0.673
Type of healthcare facility				
Conventional	447	0.36 (0.20)	Ref	–
Private for-profit	633	0.37 (0.20)	1.1 [0.9; 1.1]	0.117

95% CI, 95% confidence interval; SD, standard deviation; MR: mean ratio

Each malaria episode caused 1.2 ± 1.1 individuals (including patients and relatives) to be absent from social activities. Of 351 patients (32% of total patients) who were economically active (EA, > 15 years old), 239 (68%) were absent from daily activities (i.e., school or work) due to their illness. In addition, family members of 918 patients (85% of all patients) had to interrupt their economic activities due to patients’ requirements for malaria care. One malaria episode caused an average loss of 3.1 ± 2.2 days among EA patients and 3.4 ± 2.1 days among EA relatives [See Additional file 5]. The multivariable generalized linear model predicted that the accumulated time lost by EA patients increased substantially in patients residing in rural areas (p < 0.001), in those not covered by health insurance (p = 0.049), and those with an increased DW (p < 0.001) (Table 4).

**Table 4:** Generalized linear regression model for time losses in economically active individuals (≥ 15 years old) with uncomplicated malaria in the DRC

Variable	n	Average time (SD)	Univariate model		Multivariable final model	
			MR [95% CI]	p-value	MR [95%CI]	p-value
Gender						
Male	143	3.6 (2.7)	Ref		–	
Female	208	3.8 (2.9)	1.1 [0.9; 1.2]	0.494	–	
Age (years)	351	3.7 (4.9)	1.0 [1.0; 1.0]	0.105	1.0 [1.0; 1.0]	0.237
Residence						
Urban area	94	4.1 (1.8)	Ref		Ref	
Rural area	257	2.6 (3.2)	1.6 [1.3; 1.8]	< 0.001	1.5 [1.3; 1.8]	< 0.001
Health insurance coverage						
Yes	44	2.6 (1.9)	Ref		Ref	
No	307	3.9 (2.9)	1.5 [1.2; 1.9]	0.001	1.2 [1.0; 1.6]	0.049
Socioeconomic index quintile						
Quintile 1 "most economically disadvantaged"	55	3.9 (2.9)	Ref		–	
Quintile 2 "very economically disadvantaged"	46	4.1 (3.0)	1.0 [0.8; 1.4]	0.787	–	
Quintile 3 "economically disadvantaged"	70	4.2 (3.1)	1.1 [0.8; 1.4]	0.613	–	
Quintile 4 "less economically disadvantaged"	82	3.8 (2.8)	0.9 [0.8; 1.3]	0.844	–	
Quintile 5 "least economically disadvantaged"	98	3.1 (2.3)	0.8 [0.6; 0. 9]	0.045	–	
Type of healthcare facility						
Private for-profit	219	3.7 (2.8)	Ref		–	
Conventional	132	3.8 (2.9)	1.0 [0.9; 1.2]	0.678	–	
Disutility weight	351	3.7 (4.9)	2.8 [1.9; 4.3]	< 0.001	3.0 [2.0; 4.5]	< 0.001

95% CI, 95% confidence interval; SD, standard deviation; MR, mean ratio

### Economic costs for uncomplicated malaria episodes in the DRC

The total costs associated with 1080 uncomplicated malaria episodes in our survey were US$ 39,204: US$ 18,025 (46.0%) corresponded to direct costs and US$ 21,125 (54.0%) to indirect costs (Table 5). Average costs amounted to US$ 36.3 per episode, with US$ 16.7 being direct costs and US$ 19.6 indirect costs. As patients experienced 2.7 malaria episodes per year, the yearly cost of malaria was estimated at an average of US$ 98 per person. Indirect costs associated to the time losses due to malaria in patients (US$ 15,707) were higher than the costs for their relatives (US $5,409) while treatment costs (US$ 6,296) and follow-up costs (US$ 7,096) accounted for most of the direct costs [See Additional files 7 and 8]. The total economic costs for the DRC nationally were estimated to be US$ 503,837,635, with an uncertainty interval from US$ 492,664,040 to US$ 515,094,408 [Additional file 9].

**Table 5:** Total economic costs associated with uncomplicated malaria from the patient’s perspective, estimated from the survey in the DRC

Cost category	n	Total costs (US$)	Cost per episode (US$)	SD
Cost for all participants				
Total costs	1080	39,204	36.3	19.1
Direct costs	1080	18,025.2	16.7	11.8
Indirect costs	1080	21,124.8	19.6	12.8
Total costs by age				
Adult patients	351	14,067.1	40.1	20.2
Young patients	729	25,136.5	34.4	18.3
Total costs by area				
Rural areas	688	25,359.3	36.9	18.6
Urban areas	392	13,844.2	35.3	19.8
Total costs by facility				
Conventional healthcare facility	447	16,795.4	37.6	21.2
Private for-profit healthcare facility	633	22,408.1	35.4	17.3

The multi-way PSA, considering the total number of uncomplicated malaria episodes in DRC and accounting for the uncertainty of all relevant cost parameters at the same time, found that the uncertainty limits (2.5% and 97.5%) for the average cost per malaria episode were between US$ 35.5 and US$ 37.2 [See Additional file 9].

**Fig. 2:**
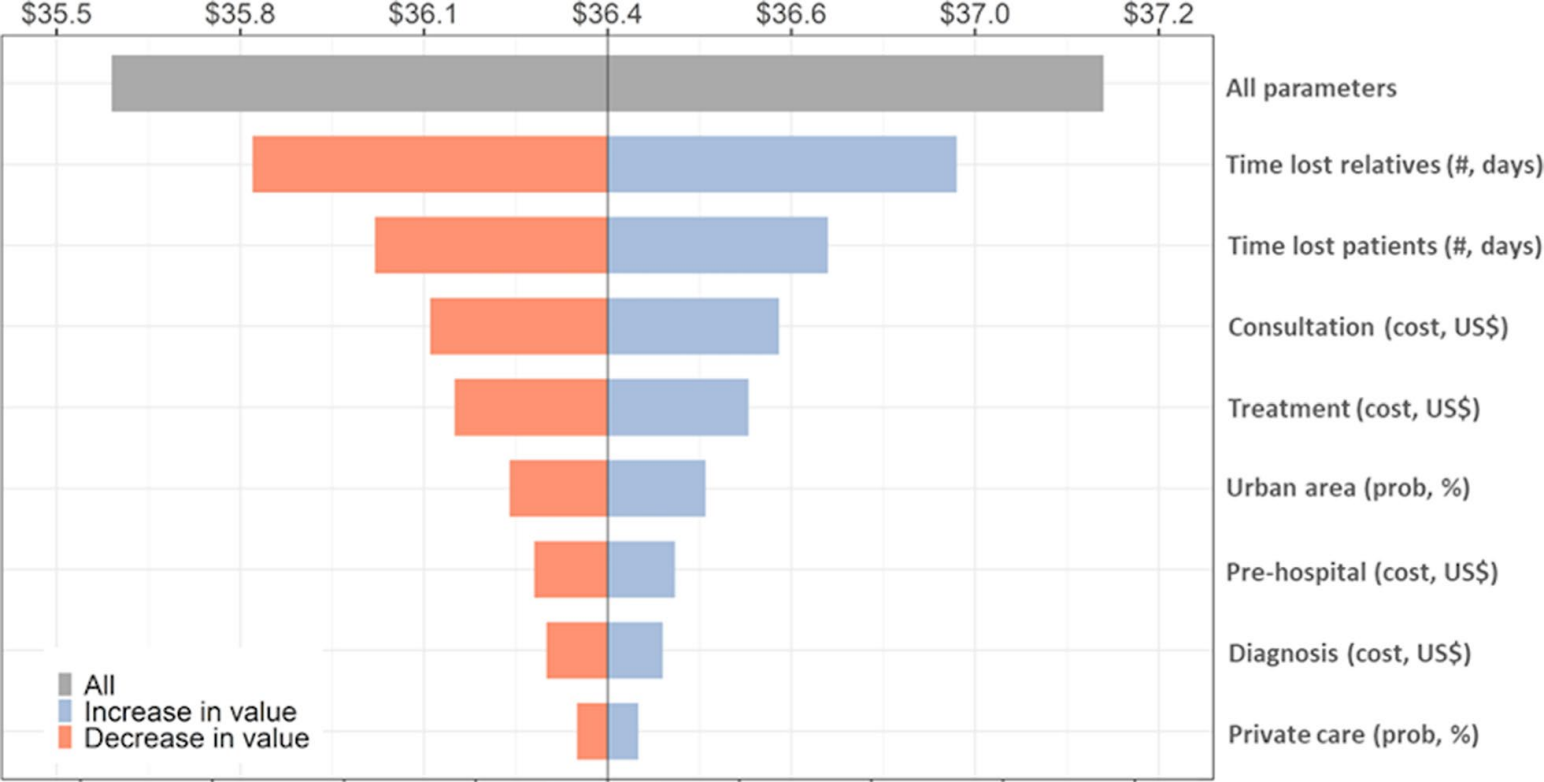
Tornado diagram of probabilistic sensitivity analysis (PSA) of the average costs per uncomplicated malaria episode in the DRC. This figure explores the effect of key parameters on the average costs per uncomplicated malaria episode in DRC, 2017. The gray bar shows the uncertainty interval of the average costs by calculating the uncertainty of all parameters (multi-way PSA). The coloured bars show the uncertainty interval of the average costs by calculating the uncertainty of one individual parameter at time (one-way PSA)

Assessing the uncertainty of individual cost parameters (one-way PSA) showed that the time lost due to malaria induced most of the uncertainty in the average cost per malaria episode, with uncertainty intervals between US$ 35.9 and US$ 36.6 when accounting for the time losses for EA patients, and US$ 35.6 and US$ 36.8 for the time losses for patients’ EA relatives (second and third coloured bars in Fig. 2). Uncertainty intervals were narrower with the initial consultation cost, treatment cost, residence area, treatment attempts at pre-hospital stage, and diagnosis cost (fourth to eighth bars in Fig. 2). The type of healthcare facility was the least influential parameter.

## Discussion

This study evaluated the socio-economic burden of uncomplicated malaria illness in the DRC, the second most-affected country in the world. Estimated total costs for the 13,863,021 uncomplicated malaria episodes registered by the NMCP in the country in 2017 were US$ 503,837,635, representing about 6% of the country’s Gross Domestic Product (GDP) for that year. Average costs per episode were US$ 36.3 (varying between US$ 35.5 and US$ 37.1), being almost 7 times higher than the national daily minimum wage. Consistent with observations in other endemic areas [4, 33–35], uncomplicated malaria leads to substantial exhaustion of family assets and loss of income, and may be an important cause of poverty in DRC. Overall, cost estimates showed limited variability. Several factors may contribute to this homogeneity of responses across uncomplicated malaria episodes. It could be the result of the narrow fringe of clinical alterations sustaining uncomplicated malaria, in comparison to severe malaria forms which are more heterogeneous in the level of severity and variability of symptoms [14]. It may also be a reflection of the regulatory efforts from the national anti-malarial policy.

More than half of the costs (54.5%) resulted from indirect costs incurred by economically active patients and related family members that were pushed to interrupt their daily activities to seek care (~ US$ 20 per episode based on minimum wage). Of 351 economically active patients (EA, > 15 years old), 239 (68%) halted their daily activities (i.e., school or work) due to illness, and of all patients, 918 (85%) had family members who interrupted their economic activities due to requirements for malaria care. In fact, one malaria episode led to a loss of about three days for economically active patients (equivalent to average US$ 15.4) and a loss of an additional three and a half days (equivalent to average US$ 17.1) accumulated in families by the patient’s relatives. Thus, as already reported in other African countries, a patient’s uncomplicated malaria putatively drives their whole family to productivity losses due to absenteeism from daily activities [15, 36, 37]. Considering the recurrence of malaria episodes in each participant (~ 3 per year) suggests the extent to which malaria contributes to the impoverishment of families in the DRC. Poverty, in turn, has the potential for generating and maintaining conditions perpetuating malaria transmission in the population [3–5, 16, 36]. Therefore, important efforts in terms of health care policies and socio-economic measures need to be implemented to break the vicious cycle between poverty and malaria that threatens the population in DRC.

The length of time lost due to illness in the current study occurred mainly at the pre-hospital stage, and this turned out to be the parameter that had the greatest influence on the average costs per malaria episode in DRC. This is understandable since consistent observations on patients with uncomplicated malaria in endemic areas have identified longer time losses as a result of delayed malaria diagnosis, potentially leading to a more compromised clinical status of patients requiring longer recovery time, and thus, higher care costs [5, 16, 34, 38–40]. Furthermore, frequent therapeutic attempts occurred at the pre-hospital stage, relying on drugs originating mainly from the informal sector (e.g., self-medication, pharmaceutical store agents, street sellers of drugs, and traditional healers) that emerged as the first choice in malaria care-seeking. In DRC, the informal sector does not benefit from the NMCP’s intervention packages (e.g., training, diagnosis, treatment) and may be a source of poor case management with risks of increased morbidity and mortality and even the emergence of drug resistance that would further increase costs associated with malaria [39, 40]. Hence, strategies aiming to reduce the time lost are probably crucial for reducing the disease economic burden. In the study population, these strategies should specifically target patients living in rural areas, those not covered by a health insurance, or those with heavy DW who were identified as categories potentially experiencing increased time loss per malaria episode.

Direct costs (45.6% of total costs) paid out of pocket by families comprised mostly treatment costs and follow-up costs. In DRC, the NMCP intervention package provides anti-malarial drugs and malaria tests free of charge for all patients reaching healthcare facilities [11, 12]. Though this is already a salutary measure for the population, healthcare policymakers should be urged to fully subsidize care for malaria as they do for other endemic infectious diseases in the country (e.g., tuberculosis, leprosy, VIH) [41]. Otherwise, an intermediate measure would be to extend the malaria care support to additional costs (e.g., fees for medical consultation). However, conversely to the rules in force, it appeared that fees were still required from patients in order to access anti-malarial drugs (around US$ 3.5 per episode) and malaria diagnosis (about US$ 0.5 per episode), which are supposed to be free of charge. Therefore, efforts for more restrictive regulation of pricing and care services to malaria patients are needed to standardize and effectively reduce direct costs paid by patients on arrival in hospitals.

Previous studies arising from different endemic countries have reported various estimates of cost-of-illness in patients with uncomplicated malaria that are similar or even significantly different from current results, ranging from US$ 180 in the Peruvian Amazon to US$ 4.9 in Ghana as total costs per episode [15, 33, 34, 42–46]. Differences noted between cost estimates can be related to applied methodological approaches as well as to regions’ related socioeconomic characteristics [33–35]. For instance, the indirect costs may substantially differ in the same country or between different countries if the estimations are based on GDP or minimum wage [34]. Malaria-associated indirect costs in the present study would have been four times lower if the estimates were based on the GDP (~ US$ 1.3 per day per capita) instead of the minimum wage (~ US$ 5 per day) [47]. Differences noted between cost estimates can also be related to inequalities in the deployment and organization of health policies between regions [33–35]. In this study, malaria-associated costs may thus have been underestimated since the collection sites in this study were selected for their relative accessibility and locations in areas with relatively high access to healthcare facilities and more intense activities of the NMCP. It is hence conceivable that in areas with more limited access to healthcare facilities, the population may be incurring a heavier malaria burden and higher cost-of-illness.

All social EQ-5D-3L dimensions (i.e., mobility, self-care, usual activities, pain/discomfort, and anxiety/depression) were moderately or severely impaired in about half of the patients with uncomplicated malaria. The DW and EQ-5D index score were estimated for assessing the magnitude of health loss associated with specific health outcomes during uncomplicated malaria. The results obtained show that one malaria episode is associated with poor self-reported quality of life and heavy disutility beside its economic burden. However, the overall average DW of 0.36 ± 0.2 noted per patient with uncomplicated malaria should be considered with caution since it cannot be definitively established as being specific to the malaria-related health condition and the starting point for each individual patient (which is not VAS = 100) was unknown. Moreover, the average EQ-5D index score of 0.62 ± 0.3 was inferred from Zimbabwean health preferences as there are not yet established Congolese population norms. A patient with uncomplicated malaria who was not benefitting from a health insurance mechanism (896 out of 1080; 82.9%) incurred substantially higher costs, lower quality of life, and heavier disutility while losing more time during illness. Health insurance is a social protection measure consisting in a mechanism of public or private policies aimed at preventing, reducing, and eliminating the economic and social vulnerabilities to poverty or deprivation resulting from disease [13]. In the DRC, only around 7% and 1% of the population residing in urban and rural areas, respectively, were found to be covered by formal health insurance programmes in 2017, mainly from employers (> 75%) [13]. Notably, wider proportions of malaria patients (4.7% in rural areas and 38.8% in urban areas) benefitted from health insurance in the current study. The difference noted would be due to mechanisms for specifically reducing malaria care costs that have been implemented by the NMCP in addition to all-disease health insurance coverage [11]. However, these coverage levels still require further improvement. In addition, the distribution of subjects holding such health insurance policies raised equity issues to the detriment of the population living in rural areas, the most economically disadvantaged, female subjects, and those with the lowest education levels. Overall, as for all other diseases in the DRC’s health system [9, 11], this study indicated that malaria health care relies heavily on patients’ direct payments despite substantial multilateral aid to the NMCP. The large population that is not covered by any health insurance mechanisms may incur catastrophic out-of-pocket expenses, especially when attending the private or informal sector for treatment. Care conditions and quality of life of malaria patients can thus be negatively affected in those not holding health insurance policies due to high health care costs that may drive the use of less effective care or practices and delay diagnosis and treatments [10, 14]. Strategies for promoting health insurance and access to malaria care services thus turn out to be crucial for reducing the disease burden in the country. In addition, patients living in rural areas incurred a disproportionally higher socioeconomic burden of uncomplicated malaria compared to those residing in urban areas as they lost more time due to illness, had limited access to health insurance, and accounted for most of the poorest patients. This observation is more likely to be indicative of the country’s health system situation. In fact, decades of non-governance have resulted in the collapse of the state and the economy in the DRC with, as correlates, a lack of public funding and weak national leadership, including within the health system [9]. This situation led the health authorities to encourage the creation of private for-profit and non-profit health care service delivery points and their integration into the health system to supply the government's inability to meet the health needs of the population [9, 10, 41]. The expansion of the private health sector is most pronounced in rural areas where the state’s facilities are fewer compared to urban areas. The NMCP thus relies heavily on private health care providers for implementing the national anti-malarial policies and related activities in different rural health districts [10, 11]. This may explain why malaria patients living in rural settings almost exclusively sought care in the private sector across this study. Furthermore, an inefficient workforce deployment is noted in the DRC’s health system with an overabundance of human resources for health in urban areas in comparison to rural areas where the population have few and distant health facilities with less qualified workers, limited delivery of services, and clinics frequently running out of materials [9]. Though integrated into the health system’s national supply, these private for-profit clinics in reality are widely unregulated and most often provide health services of unknown quality, increasing the cost of health care to the population and delaying necessary hospital referrals [10]. The efficiency and effectiveness of health care delivery is consequently affected even for freely-accessible health services such as those for care of uncomplicated malaria. It is, therefore, not surprising that the quality of life of patients with uncomplicated malaria has been more affected in rural areas. These geographical and social inequalities have been highlighted previously in the Congolese population by the EDS-RDC II 2013–2014 and require remedy by adapted social policies and public health strategies [8]. Similar situations have been reported in several African countries including Ethiopia and Mozambique [18, 48].

While increased subsidization for the management of malaria cases and better access to care services will certainly help in reducing the direct disease burden upon families, more general measures are needed to effectively reduce the cost of malaria for the country over a longer-term perspective. These measures include any control policy or prevention strategy that can contribute to reducing the number of cases and hence the costs associated with malaria. Therefore, more efforts to improve socioeconomic conditions, hygiene, and sanitation should be put forward in the country. Likewise, health communication efforts should figure prominently in strategies for reducing the socioeconomic burden of malaria via the increase of knowledge and the promotion of good practices against malaria [49]. Unsurprisingly, the vast majority of respondents had basic knowledge about malaria (route of transmission, mode of prevention, and main symptoms) and bed nets available in their respective households, especially in rural areas. This is probably because of intensive campaigns initiated in recent years throughout the country by the NMCP and community-based organizations that have been more pronounced in rural settings, likely in recognition of the extra challenges faced by patients in these regions with higher malaria prevalence [7, 11]. These awareness-raising efforts should be continued and even scaled-up in the sector of knowledge and practices that were identified to have additional gaps to be filled (e.g., patients evoking wrong transmission mechanisms, drug prescriptions from the informal sector as first choice treatments) in both rural and urban settings in this study. As the exposure to malaria is high in all settings, campaigns for improving the coverage and usage of long-lasting insecticidal bed nets (LLINs) in households must also be maintained and even strengthened in order to consolidate the performance achieved [7, 10, 50].

## Limitations

This study had some limitations. First, analyses were restricted to expenditures from a patient's perspective, which may underestimate the true cost-of-illness since this patient-centered approach does not include costs from the health care provider. For instance, expenses covered by the support from the NMCP (e.g., training of clinicians, rapid diagnostic tests, and anti-malarial drugs) were not included in cost estimates. Second, only patients with uncomplicated symptomatic malaria were considered. In cases of severe malaria, the disease burden is expected to be much heavier than that noted here because of potentially higher treatment costs, more impaired quality of life, and putative longer duration of illness with the possibility of serious or even fatal complications [14, 15, 22, 23]. Third, the study design was based on interviews with respondents in accessible NMCP sentinel sites and, therefore, cannot be spared from the memory and selection bias that this entails. Fourth, this study has provided empirical evidence based on patients reaching healthcare facilities who may be different from the general population. It is, therefore, difficult to speculate on the possible impact that this would have on the results noted in this study. The precise income lost due to illness would have strengthened the study findings. However, given the confidentiality requests made by participants, this important information could not be obtained. Cost estimations were consequently based on the minimum wage for economically active individuals, defined as those who were ≥ 15 years old. Though this empirical estimation is allowed for labour force evaluation [28], it may have biased the outcome estimates. Finally, regarding the quality of life, the population norms (i.e., in healthy people) and the baseline of patients (i.e., before the episode) were not available at the time of this study. Therefore, caution should be raised in the interpretation of HRQL problems that were noted and which could not be specifically due to the current malaria episode.

## Conclusions

Despite the above-mentioned limitations, this study highlights high cost estimates and emphasizes the considerable burden of uncomplicated malaria in the DRC. The outcomes provided will be useful to underscore the need for continued support and for soliciting appropriate funding from both government and non-government organizations for effective malaria control in the country. This study used factual information to advocate subsidizing the management of uncomplicated malaria cases through health insurance mechanisms as a key policy able to substantially reduce out-of-pocket costs for patients and their families while significantly improving the quality of their life. Categories of patients identified as associated with increased economic costs of illness in this study will be critical for decision-making, priority setting, and resource allocation of anti-malarial control policies.

## Supplementary Information

**Additional file 1:** Questionnaire.

**Additional file 2: Table S1. **Socio-economic characteristics of health insurance policy holders among patients with uncomplicated malaria in the DRC.

**Additional file 3: Table S2.** Status of malaria knowledge among patients and their respondent guardians in the DRC.

**Additional file 4: Table S3.** Vector control measures in use among malaria patients and households in the DRC.

**Additional file 5: Table S4.** Trajectory followed by patients for malaria care-seeking in the DRC.

**Additional file 6: Table S5.** EQ-5D-3L health related quality of life during malaria episode in the DRC.

**Additional file 7: Table S6.** Indirect costs of uncomplicated malaria estimated through the survey in the DRC.

**Additional file 8: Table S7.** Direct costs of uncomplicated malaria estimated through the survey in the DR.C

**Additional file 9: Table S8.** Parameters, base case estimates, and uncertainty distribution for probabilistic analyses of economic costs of uncomplicated malaria in the DRC.

## Data Availability

All data generated during this study are included in the published article and its Aditional files.

## Abbreviations

DRC: Democratic Republic of Congo
NMCP: National Malaria Control Programme
EQ-5D-3L: EuroQol Group’s 5 dimensions descriptive system
EQ VAS: EuroQol Group’s visual analogue scale
PSA: Probabilistic sensitivity analyses
DW: Disutility weight
RDT: Rapid Diagnostic Test
GDP: Gross Domestic Product
HD: Health District
GRH: General Reference Hospital
SNIS: National Health Information System
HRQL: Health-related quality of life
SD: Standard Deviation
SE: Standard Error
GLMs: Generalized Linear Models
