# Supplementary material for: Care-seeking behaviour and socio-economic burden associated with uncomplicated malaria in the Democratic Republic of Congo
Source: Malar J. 2021 Jun 9;20:260. doi: 10.1186/s12936-021-03789-w (PMC8191196; doi:10.1186/s12936-021-03789-w)
Supplement: Supplementary file 2 — Additional file 2: Table S1. Socio-economic characteristics of health insurance policy holders among patients with uncomplicated malaria in the DRC. [file 12936_2021_3789_MOESM2_ESM.docx]

# **Additional file 2: Table S1. Socio-economic characteristics of health insurance policy holders among patients with uncomplicated malaria in the DRC**

| **Characteristics** | | **Health insurance policy holder** | | | | **p-value** |
| --- | --- | --- | --- | --- | --- | --- |
|  |  | **Yes** | | **No** | |  |
|  |  | **n** | **%** | **n** | **%** |  |
| **Gender** | |  |  |  |  |  |
|  | Female | 79 | 14.3 | 474 | 85.7 | Ref. |
|  | Male | 105 | 19.9 | 422 | 80.1 | 0.017 |
| **Age group** | |  |  |  |  |  |
|  | Adult patients | 44 | 12.5 | 307 | 87.5 | Ref |
|  | Young patients | 140 | 19.2 | 589 | 80.8 | 0.008 |
| **Education level (n=257; age ≥18 years)** | |  |  |  |  |  |
|  | No education | 0 | 0.0 | 38 | 100.0 | Ref. |
|  | Primary level | 3 | 6.8 | 41 | 93.2 | 0.241 |
|  | High school level | 21 | 13.0 | 140 | 87.0 | 0.016 |
|  | College or University level | 12 | 27.3 | 32 | 72.7 | 0.002 |
| **Area** | |  |  |  |  |  |
|  | Rural area | 32 | 4.7 | 656 | 95.3 | Ref. |
|  | Urban area | 152 | 38.8 | 240 | 61.2 | <0.001 |
| **Socio-economic category** | |  |  |  |  |  |
|  | Quintile 1 "most economically disadvantaged" | 2 | 0.9 | 217 | 99.1 | Ref. |
|  | Quintile 2 "very economically disadvantaged" | 59 | 26.9 | 160 | 73.1 | <0.001 |
|  | Quintile 3 "economically disadvantaged" | 26 | 12.0 | 190 | 88.0 | <0.001 |
|  | Quintile 4 "less economically disadvantaged" | 37 | 16.9 | 182 | 83.1 | <0.001 |
|  | Quintile 5 "least economically disadvantaged" | 60 | 29.0 | 147 | 71.0 | <0.001 |
| **Type of healthcare facility** | |  |  |  |  |  |
|  | Conventional | 106 | 25.9 | 303 | 74.1 | Ref. |
|  | Private for-profit | 78 | 11.6 | 593 | 88.4 | <0.001 |
| **All patients** | |  |  |  |  |  |
|  | Patients | 184 | 17.0 | 896 | 83.0 | - |
