# Supplementary material for: Care-seeking behaviour and socio-economic burden associated with uncomplicated malaria in the Democratic Republic of Congo
Source: Malar J. 2021 Jun 9;20:260. doi: 10.1186/s12936-021-03789-w (PMC8191196; doi:10.1186/s12936-021-03789-w)
Supplement: Supplementary file 3 — Additional file 3: Table S2. Status of malaria knowledge among patients and their respondent guardians in the DRC. [file 12936_2021_3789_MOESM3_ESM.docx]

# **Additional file 3: Table S2. Status of malaria knowledge among patients and their respondent guardians in the DRC**

| **Characteristics** | | **Rural area** | | **Urban area** | | **Total** | | **p-value** |
| --- | --- | --- | --- | --- | --- | --- | --- | --- |
|  |  | **n=688** | | **n=392** | | **n=1080** | |  |
|  |  | **n** | **%** | **n** | **%** | **n** | **%** |  |
| **Evoked mechanisms of transmission of malaria** | |  |  |  |  |  |  |  |
|  | Mosquito bite | 587 | 85.3 | 310 | 79.1 | 897 | 83.1 | 0.011 |
|  | Unknown | 50 | 7.3 | 27 | 6.9 | 77 | 7.1 | 0.912 |
|  | Non-sanitized environment | 3 | 0.4 | 68 | 17.3 | 71 | 6.6 | <0.001 |
|  | Insect bites | 26 | 3.8 | 15 | 3.8 | 41 | 3.8 | 1.000 |
|  | Blood transfusion | 29 | 4.2 | 11 | 2.8 | 40 | 3.7 | 0.312 |
|  | Drinking water | 40 | 5.8 | 24 | 6.1 | 64 | 5.9 | 0.942 |
|  | Contaminated foods | 10 | 1.5 | 15 | 3.8 | 25 | 2.3 | 0.022 |
|  | Transmission from mother to baby | 22 | 3.2 | 2 | 0.5 | 24 | 2.2 | 0.008 |
|  | Witchcraft | 7 | 1.0 | 2 | 0.5 | 9 | 0.8 | 0.594 |
|  | Corporal hygiene | 2 | 0.3 | 1 | 0.3 | 3 | 0.3 | 1.000 |
| **Evoked symptoms of malaria (n=921)** | |  |  |  |  |  |  |  |
|  | Fever | 586 | 85.2 | 196 | 84.1 | 782 | 84.9 | 0.777 |
|  | Headache | 350 | 50.9 | 86 | 36.9 | 436 | 47.3 | <0.001 |
|  | Chills | 214 | 31.1 | 97 | 41.6 | 311 | 33.8 | 0.004 |
|  | Generalized pain | 170 | 24.7 | 35 | 15 | 205 | 22.3 | 0.003 |
|  | Vomiting | 78 | 11.3 | 49 | 21 | 127 | 13.8 | <0.001 |
|  | Convulsions | 96 | 14 | 14 | 6 | 110 | 11.9 | 0.002 |
|  | Diarrhoea | 58 | 8.4 | 15 | 6.4 | 73 | 7.9 | 0.405 |
|  | Asthenia | 29 | 4.2 | 14 | 6 | 43 | 4.7 | 0.346 |
|  | Anorexia | 10 | 1.5 | 9 | 3.9 | 19 | 2.1 | 0.049 |
|  | Coma | 12 | 1.7 | 0 | 0 | 12 | 1.3 | 0.09 |
| **Evoked method for preventing malaria** | |  |  |  |  |  |  |  |
|  | Mosquito net impregnated with insecticide | 474 | 68.9 | 155 | 39.5 | 629 | 58.2 | <0.001 |
|  | Simple mosquito net | 136 | 19.8 | 147 | 37.5 | 283 | 26.2 | <0.001 |
|  | Sanitized environment | 130 | 18.9 | 85 | 21.7 | 215 | 19.9 | 0.27 |
|  | Spreading insecticides | 47 | 6.8 | 51 | 13 | 98 | 9.1 | 0.001 |
|  | No protection against mosquitos exists | 41 | 6 | 38 | 9.7 | 79 | 7.3 | 0.032 |
|  | Smoke sticks | 47 | 6.8 | 51 | 13 | 98 | 9.1 | 0.001 |
|  | Vegetal oils | 0 | 0.0 | 3 | 0.8 | 3 | 0.3 | 0.021 |
|  | Medicinal plants | 1 | 0.1 | 0 | 0.0 | 1 | 0.1 | <0.001 |
|  | Don't know | 10 | 1.5 | 2 | 0.5 | 12 | 1.1 | 0.155 |
