# Supplementary material for: Care-seeking behaviour and socio-economic burden associated with uncomplicated malaria in the Democratic Republic of Congo
Source: Malar J. 2021 Jun 9;20:260. doi: 10.1186/s12936-021-03789-w (PMC8191196; doi:10.1186/s12936-021-03789-w)
Supplement: Supplementary file 4 — Additional file 4: Table S3. Vector control measures in use among malaria patients and households in the DRC. [file 12936_2021_3789_MOESM4_ESM.docx]

# **Additional file 4: Table S3. Vector control measures in use among malaria patients and households in the DRC**

| **Characteristics** | | **Rural area** | | **Urban area** | | **Total** | | **p-value** |
| --- | --- | --- | --- | --- | --- | --- | --- | --- |
|  |  | **n=688** | | **n=392** | | **n=1080** | |  |
|  |  | **N** | **%** | **N** | **%** | **n** | **%** |  |
| **Availability of a bed net in the household** | |  |  |  |  |  |  | 0.468 |
|  | No | 61 | 8.9 | 40 | 10.2 | 101 | 9.4 |  |
|  | Yes | 627 | 91.1 | 352 | 89.8 | 979 | 90.6 |  |
| **Nature of bed net available in the household (n=979)** | |  |  |  |  |  |  | 0.003 |
|  | Bed net with insecticide | 610 | 97.3 | 329 | 93.5 | 939 | 95.9 |  |
|  | Bed net without insecticide | 17 | 2.7 | 23 | 6.5 | 40 | 4.1 |  |
| **Time since the acquisition of the bed nets in the household (n=935)** | |  |  |  |  |  |  | 0.001 |
|  | Less than 3 years | 552 | 93.2 | 336 | 98.0 | 888 | 95.0 |  |
|  | More than 3 years | 40 | 6.8 | 7 | 2.0 | 47 | 5.0 |  |
| **Last night spent at home under a bed net (n=979)** | |  |  |  |  |  |  | 0.730 |
|  | No | 144 | 23 | 84 | 23.9 | 228 | 23.3 |  |
|  | Yes | 483 | 77 | 268 | 76.1 | 751 | 76.7 |  |
| **Last night spent at home under a bed net by patients aged under 5 years (n=356)** | |  |  |  |  |  |  | 0.458 |
|  | No | 41 | 18.5 | 24 | 17.9 | 65 | 18.3 |  |
|  | Yes | 181 | 81.5 | 110 | 82.1 | 291 | 81.7 |  |
| **Number of bed nets in the household (n=979)** | |  |  |  |  |  |  | 0.252 |
|  | More than one | 505 | 80.5 | 297 | 84.4 | 802 | 81.9 |  |
|  | One | 122 | 19.5 | 55 | 15.6 | 177 | 18.1 |  |
| **Spraying of insecticide during the last 12 months** | |  |  |  |  |  |  | <0.001 |
|  | No | 627 | 91.1 | 381 | 97.2 | 1008 | 93.3 |  |
|  | Yes | 61 | 8.9 | 11 | 2.8 | 72 | 6.7 |  |
