# Supplementary material for: Care-seeking behaviour and socio-economic burden associated with uncomplicated malaria in the Democratic Republic of Congo
Source: Malar J. 2021 Jun 9;20:260. doi: 10.1186/s12936-021-03789-w (PMC8191196; doi:10.1186/s12936-021-03789-w)
Supplement: Supplementary file 5 — Additional file 5: Table S4. Trajectory followed by patients for malaria care-seeking in the DRC. [file 12936_2021_3789_MOESM5_ESM.docx]

# **Additional file 5: Table S4. Trajectory followed by patients for malaria care-seeking in the DRC**

| **Characteristics^§^** | | | **Rural area** | | **Urban area** | | **Total** | | **p-value** |
| --- | --- | --- | --- | --- | --- | --- | --- | --- | --- |
|  |  |  | **n=688** | | **n=392** | | **n=1080** | |  |
|  |  |  | **n** | **%** | **n** | **%** | **n** | **%** |  |
| **Previous malaria episodes** | | |  |  |  |  |  |  |  |
|  | **Malaria episode experienced during the last 12 months** | | | | |  |  |  | 0.041 |
|  |  | No | 294 | 42.7 | 156 | 39.8 | 450 | 41.7 |  |
|  |  | Yes | 393 | 57.1 | 231 | 58.9 | 624 | 57.8 |  |
|  |  | Do not know | 1 | 0.1 | 5 | 1.3 | 6 | 0.6 |  |
|  | **Number of malaria episodes reported over one year (n=630)*** | | | | | |  |  | 0.602 |
|  |  | More than three | 89 | 22.6 | 58 | 24.6 | 147 | 23.3 |  |
|  |  | One to three | 297 | 75.4 | 171 | 72.5 | 468 | 74.3 |  |
|  |  | Do not know | 8 | 2.0 | 7 | 3.0 | 15 | 2.4 |  |
|  | **Time passed since the last malaria episode (n=630)** ^†^ | | | |  |  |  |  | 0.051 |
|  |  | Less than 3 months | 167 | 42.4 | 106 | 44.9 | 273 | 43.3 |  |
|  |  | 3 to 6 months | 119 | 30.2 | 71 | 30.1 | 190 | 30.2 |  |
|  |  | More than 6 months | 69 | 17.5 | 50 | 21.2 | 119 | 18.9 |  |
|  |  | Do not know | 39 | 9.9 | 9 | 3.8 | 48 | 7.6 |  |
|  | **Laboratory test performed in the last malaria episode (n=630)** | | | | | |  |  | 0.102 |
|  |  | No | 65 | 16.5 | 55 | 23.3 | 120 | 19 |  |
|  |  | Yes | 321 | 81.5 | 174 | 73.7 | 495 | 78.6 |  |
|  |  | Do not know | 8 | 2.0 | 7 | 3.0 | 15 | 2.4 |  |
|  | **Antimalarial medications taken in the last malaria episode (n=630)** | | | | | |  |  | 0.005 |
|  |  | No | 34 | 8.6 | 8 | 3.4 | 42 | 6.7 |  |
|  |  | Yes | 359 | 91.1 | 223 | 94.5 | 582 | 92.4 |  |
|  |  | Do not know | 1 | 0.3 | 5 | 2.1 | 6 | 1.0 |  |
|  | **Antimalarial treatment taken in the last malaria episode (n=630)** | | | | | | |  | <0.001 |
|  |  | ACT | 263 | 66.8 | 111 | 47.0 | 374 | 59.4 |  |
|  |  | Artesunate monotherapy | 2 | 0.5 | 0 | 0.0 | 2 | 0.3 |  |
|  |  | Artemether inj. | 8 | 2.0 | 13 | 5.5 | 21 | 3.3 |  |
|  |  | Do not know | 1 | 0.3 | 12 | 5.1 | 13 | 2.1 |  |
|  |  | Medicinal plants | 1 | 0.3 | 0 | 0.0 | 1 | 0.2 |  |
|  |  | Quinine | 61 | 15.5 | 76 | 32.2 | 137 | 21.7 |  |
|  |  | SP | 7 | 1.8 | 5 | 2.1 | 12 | 1.9 |  |
|  |  | No treatment | 30 | 7.6 | 11 | 4.7 | 41 | 6.5 |  |
|  | **Origin of antimalarial drugs taken in the last malaria episode (n=630)** | | | | | | |  | 0.008 |
|  |  | Pharmaceutical store agent/street vendor of pharmaceutical products/traditional healer/patients relatives/the patient himself | 64 | 16.2 | 59 | 25 | 123 | 19.5 |  |
|  |  | Prescription from a health care worker from a well-known health facility | 285 | 72.3 | 163 | 69.1 | 448 | 71.1 |  |
|  |  | Do not know | 45 | 11.4 | 14 | 5.9 | 59 | 9.4 |  |
| **Current malaria episode** | | |  |  |  |  |  |  |  |
|  | **Medications taken at the pre-hospital stage** | | | |  |  |  |  | <0.001 |
|  |  | No | 244 | 35.5 | 91 | 23.2 | 335 | 31 |  |
|  |  | Yes | 444 | 64.5 | 298 | 76 | 742 | 68.7 |  |
|  |  | Do not know | 0 | 0.0 | 3 | 0.8 | 3 | 0.3 |  |
|  | **Types of antimalarial treatment received before hospital (n=745)** | | | | | |  |  | 1.000 |
|  |  | ACT | 50 | 11.3 | 4 | 1.3 | 54 | 7.2 |  |
|  |  | No antimalarial drug | 293 | 66 | 256 | 85 | 549 | 73.7 |  |
|  |  | Quinine | 49 | 11 | 19 | 6.3 | 68 | 9.1 |  |
|  |  | Unknown | 29 | 6.5 | 19 | 6.3 | 48 | 6.4 |  |
|  |  | Sulfadoxine-Pyrimethamine | 9 | 2 | 1 | 0.3 | 10 | 1.3 |  |
|  |  | Artemether inj. | 6 | 1.4 | 2 | 0.7 | 8 | 1.1 |  |
|  |  | Artesunate monotherapy | 6 | 1.4 | 0 | 0 | 6 | 0.8 |  |
|  |  | Amodiaquine monotherapy | 1 | 0.2 | 0 | 0 | 1 | 0.1 |  |
|  |  | Malarone | 1 | 0.2 | 0 | 0 | 1 | 0.1 |  |
|  | **Origin of antimalarial drugs taken in the last malaria episode (n=745)** | | | | | | |  | 0.007 |
|  |  | Prescription from the informal sector^$^ | 332 | 74.8 | 202 | 67.1 | 534 | 71.7 |  |
|  |  | Prescription from a health care worker from a well-known facility | 82 | 18.5 | 81 | 26.9 | 163 | 21.9 |  |
|  |  | Do not know | 30 | 6.8 | 18 | 6.0 | 48 | 6.4 |  |
|  | **Vitamin supplementation (n=745)** | | |  |  |  |  |  | <0.001 |
|  |  | No | 394 | 88.7 | 272 | 90.4 | 666 | 89.4 |  |
|  |  | Yes | 21 | 4.7 | 10 | 3.3 | 31 | 4.2 |  |
|  |  | Do not know | 29 | 6.5 | 19 | 6.3 | 48 | 6.4 |  |
|  | **Antipyretic or analgesic medication (n=745)** | | | |  |  |  |  | <0.001 |
|  |  | No | 92 | 20.7 | 16 | 5.3 | 108 | 14.5 |  |
|  |  | Yes | 323 | 72.7 | 266 | 88.4 | 589 | 79.1 |  |
|  |  | Do not know | 29 | 6.5 | 19 | 6.3 | 48 | 6.4 |  |
|  | **Antibiotic therapy (n=745)** | |  |  |  |  |  |  | <0.001 |
|  |  | No | 354 | 79.7 | 269 | 89.4 | 623 | 83.6 |  |
|  |  | Yes | 61 | 13.7 | 13 | 4.3 | 74 | 9.9 |  |
|  |  | Do not know | 29 | 6.5 | 19 | 6.3 | 48 | 6.4 |  |
|  | **Medicinal herbs (n=745)** | |  |  |  |  |  |  | <0.001 |
|  |  | No | 412 | 92.8 | 279 | 92.7 | 691 | 92.8 |  |
|  |  | Yes | 3 | 0.7 | 3 | 1 | 6 | 0.8 |  |
|  |  | Do not know | 29 | 6.5 | 19 | 6.3 | 48 | 6.4 |  |
|  | **Antihelminthic drugs (n=745)** | | |  |  |  |  |  | 0.001 |
|  |  | No | 404 | 91 | 274 | 91 | 678 | 91 |  |
|  |  | Yes | 11 | 2.5 | 8 | 2.7 | 19 | 2.6 |  |
|  |  | Do not know | 29 | 6.5 | 19 | 6.3 | 48 | 6.4 |  |
|  | **Time spent at pre-hospital stage**^¥^ | | |  |  |  |  |  | 0.093 |
|  |  | ≤1 day | 112 | 16.3 | 49 | 12.5 | 161 | 14.9 |  |
|  |  | >1days | 576 | 83.7 | 343 | 87.5 | 919 | 85.1 |  |
|  | (*) Average number of malaria episodes reported over one year (episodes per person): 2.72±0.05; (^†^) Average time passed since the last malaria episode (year per person): 0.31±0.23; (^¥^) Average time spent at pre-hospital stage (days per person): 3.27±0.05; (^$^) Informal sector: Pharmaceutical store agent/street vendor of pharmaceutical products/traditional healer/patients relatives/the patient himself; (**^§^**) other characteristics: average number of episodes per year (episodes): 2.72±1.64; average duration of illness (days per episode): 6.28±1.97; average duration of illness at pre-hospital stage (days per episode): 3.28±1.97. | | | | | | | | |
