# Supplementary material for: Care-seeking behaviour and socio-economic burden associated with uncomplicated malaria in the Democratic Republic of Congo
Source: Malar J. 2021 Jun 9;20:260. doi: 10.1186/s12936-021-03789-w (PMC8191196; doi:10.1186/s12936-021-03789-w)
Supplement: Supplementary file 6 — Additional file 6: Table S5. EQ-5D-3L health related quality of life during malaria episode in the DRC. [file 12936_2021_3789_MOESM6_ESM.docx]

# **Additional file 6: Table S5. EQ-5D-3L health related quality of life during malaria episode in the DRC**

| **EQ-5D-3L Dimension and related alteration** | | | **Rural area** | | | | **Urban area** | | | | **Total** | | | | **p-value** | |  |
| --- | --- | --- | --- | --- | --- | --- | --- | --- | --- | --- | --- | --- | --- | --- | --- | --- | --- |
|  |  |  | **n** | | **%** | | **n** | | **%** | | **n** | | **%** | |  |  |  |
| **Mobility** | | |  | |  | |  | |  | |  | |  | | 0.08 | |  |
|  | Altered | | | 348 | | 50.6 | | 220 | | 56.1 | | 568 | | 52.6 | |  | |
|  | Normal | | | 340 | | 49.4 | | 172 | | 43.9 | | 512 | | 47.4 | |  | |
| **Self care** | |  | | |  | |  | |  | |  | |  | | <0.001 | |  |
|  | Altered | | | 375 | | 54.5 | | 254 | | 64.8 | | 629 | | 58.2 | |  | |
|  | Normal | | | 313 | | 45.5 | | 138 | | 35.2 | | 451 | | 41.8 | |  | |
| **Usual activity** | |  | | |  | |  | |  | |  | |  | | <0.001 | |  |
|  | Altered | | | 463 | | 67.3 | | 318 | | 81.1 | | 781 | | 72.3 | |  | |
|  | Normal | | | 225 | | 32.7 | | 74 | | 18.9 | | 299 | | 27.7 | |  | |
| **Pain or discomfort** | |  | | |  | |  | |  | |  | |  | | 0.43 | |  |
|  | Altered | | | 510 | | 74.1 | | 299 | | 76.3 | | 809 | | 74.9 | |  | |
|  | Normal | | | 178 | | 25.9 | | 93 | | 23.7 | | 271 | | 25.1 | |  | |
| **Anxiety or depression** | |  | | |  | |  | |  | |  | |  | | <0.001 | |  |
|  | Altered | | | 367 | | 53.3 | | 289 | | 73.7 | | 656 | | 60.7 | |  | |
|  | Normal | | | 321 | | 46.7 | | 103 | | 26.3 | | 424 | | 39.3 | |  | |
