# Supplementary material for: Care-seeking behaviour and socio-economic burden associated with uncomplicated malaria in the Democratic Republic of Congo
Source: Malar J. 2021 Jun 9;20:260. doi: 10.1186/s12936-021-03789-w (PMC8191196; doi:10.1186/s12936-021-03789-w)
Supplement: Supplementary file 7 — Additional file 7: Table S6. Indirect costs of uncomplicated malaria estimated through the survey in the DRC. [file 12936_2021_3789_MOESM7_ESM.docx]

# **Additional file 7: Table S6. Indirect costs of uncomplicated malaria estimated through the survey in the DRC**

| **Cost category** | | | |  | **n** | **Total costs (US$)** | **Average Cost per episode (US$)** | **SD** |
| --- | --- | --- | --- | --- | --- | --- | --- | --- |
| Total indirect costs per episode | | | |  |  |  |  |  |
|  | Total indirect costs for all patients | | | | 1080 | 21235 | 19.6 | 12.8 |
| Total indirect costs per episode by site | | | | |  |  |  |  |
|  | Urban areas | | | |  |  |  |  |
|  |  | Adult patients |  |  | 94 | 1760 | 18.7 | 13.2 |
|  |  | Young patients | |  | 298 | 5025 | 16.9 | 8.9 |
|  | Subtotal | |  |  | 392 | 6785 | 17.3 | 10.1 |
|  | Rural areas | | | |  |  |  |  |
|  |  | Adult patients |  |  | 257 | 6250 | 24.3 | 16.1 |
|  |  | Young patients | |  | 431 | 8200 | 19.0 | 12.1 |
|  | Subtotal | |  |  | 688 | 14450 | 21.0 | 13.0 |
| Total indirect costs per episode by age group | | | | |  |  |  |  |
|  | Adult patients | | | |  |  |  |  |
|  |  | Rural areas | |  | 257 | 6250 | 24.3 | 16.1 |
|  |  | Urban areas | |  | 94 | 1760 | 18.7 | 13.2 |
|  | Subtotal | |  |  | 351 | 8010 | 22.8 | 15.5 |
|  | Young patients | | | |  |  |  |  |
|  |  | Rural areas | |  | 431 | 8200 | 19.0 | 12.1 |
|  |  | Urban areas | |  | 298 | 5025 | 16.9 | 8.9 |
|  | Subtotal | |  |  | 729 | 13225 | 18.1 | 10 |
| Indirect cost incurred by an EA patient during one episode | | | | |  |  |  |  |
|  | All patients | |  |  | 351 | 5410 | 15.4 | 11.3 |
| Costs in different age groups | | |  |  |  |  |  |  |
|  |  | Adult patients |  |  | 351 | 5410 | 15.4 | 11.3 |
|  |  | Young patients | |  | - | - | - | - |
| Costs by site | | |  |  |  |  |  |  |
|  |  | Rural area |  |  | 257 | 4420 | 17.2 | 11.4 |
|  |  | Urban area |  |  | 94 | 990 | 10.5 | 9.2 |
| Costs by type of healthcare facility | | | |  |  |  |  |  |
|  |  | Conventional | |  | 132 | 1815 | 13.8 | 10.2 |
|  |  | Private for-profit | |  | 219 | 3595 | 16.4 | 11.8 |
| Indirect cost incurred by a patient's EA relatives during one episode | | | | |  |  |  |  |
|  | All patients | |  |  | 918 | 15710 | 17.1 | 10.7 |
|  | Costs by age groups | | |  |  |  |  |  |
|  |  | Adult patients |  |  | 199 | 2535 | 12.7 | 8.7 |
|  |  | Young patients | |  | 719 | 13175 | 18.3 | 10.9 |
| Costs by types of site | | |  |  |  |  |  |  |
|  |  | Rural area |  |  | 561 | 9965 | 17.8 | 11.6 |
|  |  | Urban area |  |  | 357 | 5745 | 16.1 | 9.1 |
| Costs by type of healthcare facility | | | |  |  |  |  |  |
|  |  | Conventional | |  | 395 | 7650 | 19.4 | 13.1 |
|  |  | Private for-profit | |  | 523 | 8060 | 15.4 | 8.0 |
