# Supplementary material for: Care-seeking behaviour and socio-economic burden associated with uncomplicated malaria in the Democratic Republic of Congo
Source: Malar J. 2021 Jun 9;20:260. doi: 10.1186/s12936-021-03789-w (PMC8191196; doi:10.1186/s12936-021-03789-w)
Supplement: Supplementary file 8 — Additional file 8: Table S7. Direct costs of uncomplicated malaria estimated through the survey in the DR.C [file 12936_2021_3789_MOESM8_ESM.docx]

# **Additional file 8: Table S7. Direct costs of uncomplicated malaria estimated through the survey in the DRC**

| **Cost category** | | | | **n** | **Total costs (US$)** | **Average cost per episode (US$)** | **SD** |
| --- | --- | --- | --- | --- | --- | --- | --- |
| Total direct cost per episode | | | |  |  |  |  |
| Total direct cost for all patients | | | | 1080 | 18022.5 | 16.7 | 11.8 |
| Cost of medications and care expenses at the pre-hospital stage | | | |  |  |  |  |
|  |  | Rural area | | 688 | 471.5 | 0.7 | 0.7 |
|  |  | Urban area | | 392 | 176.4 | 0.5 | 0.5 |
|  | Subtotal | | | 1080 | 0.6 | 0.6 | 1.8 |
| Cost of initial consultation | | | |  |  |  |  |
|  | Conventional healthcare facility | | |  |  |  |  |
|  |  | Adult patients | | 132 | 340.6 | 2.6 | 2.6 |
|  |  | Young patients | | 315 | 941.9 | 2.9 | 3.0 |
|  | Private for-profit healthcare facility | | |  |  |  |  |
|  |  | Adult patients | | 219 | 512.5 | 2.3 | 2.3 |
|  |  | Young patients | | 414 | 931.5 | 2.3 | 2.3 |
|  | Subtotal | |  | 1080 | 2771.3 | 2.6 | 2.6 |
| Cost of laboratory tests | | | |  |  |  |  |
|  | Rural area | | |  |  |  |  |
|  |  | Conventional healthcare facility | |  |  |  |  |
|  |  |  | Adult patients | 103 | 67.7 | 0.7 | 1.2 |
|  |  |  | Young patients | 152 | 97.9 | 0.6 | 1.9 |
|  |  | Private for-profit healthcare facility | |  |  |  |  |
|  |  |  | Adult patients | 154 | 73.3 | 0.5 | 1.5 |
|  |  |  | Young patients | 279 | 120.8 | 0.4 | 1.2 |
|  | Urban area | | |  |  |  |  |
|  |  | Conventional healthcare facility | |  |  |  |  |
|  |  |  | Adult patients | 29 | 0.0 | 0.0 | 0.0 |
|  |  |  | Young patients | 163 | 2.1 | 0.0 | 0.1 |
|  |  | Private for-profit healthcare facility | |  |  |  |  |
|  |  |  | Adult patients | 65 | 79 | 1.2 | 2.4 |
|  |  |  | Young patients | 135 | 149.9 | 1.1 | 2.7 |
|  | Subtotal | |  | 1080 | 591.6 | 0.6 | 0.6 |
| Cost of malaria drug | | | |  |  |  |  |
|  | Rural area | | |  |  |  |  |
|  |  | Conventional healthcare facility | |  |  |  |  |
|  |  |  | Adult patients | 103 | 398.6 | 3.9 | 2.7 |
|  |  |  | Young patients | 152 | 592.8 | 3.9 | 3.3 |
|  |  | Private for-profit healthcare facility | |  |  |  |  |
|  |  |  | Adult patients | 154 | 585.2 | 3.8 | 2.7 |
|  |  |  | Young patients | 279 | 1090.9 | 3.9 | 2.3 |
|  | Urban area | | |  |  |  |  |
|  |  | Conventional healthcare facility | |  |  |  |  |
|  |  |  | Adult patients | 29 | 102.7 | 3.5 | 2.4 |
|  |  |  | Young patients | 163 | 223.3 | 1.4 | 2.8 |
|  |  | Private for-profit healthcare facility | |  |  |  |  |
|  |  |  | Adult patients | 65 | 445.3 | 6.9 | 8.7 |
|  |  |  | Young patients | 135 | 827.6 | 6.1 | 6.5 |
|  | Subtotal | |  | 1080 | 4267.8 | 4.0 | 4.3 |
| Cost of care services and administration | | | |  |  |  |  |
|  | All patients | | | 1080 | 2028.8 | 1.9 | 4.3 |
| Cost of transport for patient and related companions | | | |  |  |  |  |
|  | Rural area | | | 688 | 306.4 | 0.5 | 2.1 |
|  | Urban area | | | 392 | 299 | 0.8 | 3.6 |
|  | Subtotal | |  | 1080 | 605.4 | 0.6 | 2.3 |
| Cost of other non-medical fees (communication, food, etc.,) | | | |  |  |  |  |
|  | Rural area | | | 688 | 9.1 | 0.0 | 0.2 |
|  | Urban area | | | 392 | 10.6 | 0.0 | 0.3 |
|  | Subtotal | |  | 1080 | 19.7 | 0.0 | 0.3 |
| Cost of medical follow-up | | | |  |  |  |  |
|  | Conventional healthcare facility | | |  |  |  |  |
|  |  | Adult patients | | 132 | 1028.3 | 7.8 | 2.5 |
|  |  | Young patients | | 315 | 2085.3 | 6.6 | 3.0 |
|  | Private for-profit healthcare facility | | |  |  |  |  |
|  |  | Adult patients | | 219 | 1388.5 | 6.3 | 2.4 |
|  |  | Young patients | | 414 | 2587.5 | 6.3 | 2.0 |
|  | Subtotal | |  | 1080 | 7091.3 | 6.6 | 2.5 |
