# Supplementary material for: Care-seeking behaviour and socio-economic burden associated with uncomplicated malaria in the Democratic Republic of Congo
Source: Malar J. 2021 Jun 9;20:260. doi: 10.1186/s12936-021-03789-w (PMC8191196; doi:10.1186/s12936-021-03789-w)
Supplement: Supplementary file 9 — Additional file 9: Table S8. Parameters, base case estimates, and uncertainty distribution for probabilistic analyses of economic costs of uncomplicated malaria in the DRC. [file 12936_2021_3789_MOESM9_ESM.docx]

# **Additional file 9 : Table S8. Parameters, base case estimates, and uncertainty distribution for probabilistic analyses of economic costs of uncomplicated malaria in the DRC**

| **Parameter** | **Distribution** | **Population /sample size** | **Base case**  **(mean, proportion)** | **Standard errors**  **(SE)** |
| --- | --- | --- | --- | --- |
| Malaria in DRC in 2017, episodes | - | 13,863,021 | - | - |
| Malaria in 10 collection sites, episodes | - | 1080 | - | - |
| Economically active (EA) patient, probability | Beta | 1080 | 0.32 | 0.02 |
| Patient living in urban area, probability | Beta | 1080 | 0.36 | 0.02 |
| Patients with private care, probability | Beta | 1080 | 0.59 | 0.02 |
| Time lost by EA patients, person-days * | Gamma | 351 | 3.08 | 0.12 |
| Time lost by a patient's EA relatives, person-days * | Gamma | 918 | 3.42 | 0.07 |
| Cost of medications and care expenses at the pre-hospital stage, US$ * | Gamma | 1080 | 0.60 | 0.05 |
| Cost of consultation, US$ * | Gamma | 1080 | 2.57 | 0.08 |
| Cost of diagnostic test, US$ * | Gamma | 1080 | 0.55 | 0.05 |
| Cost of treatment with antimalarials, US$ * | Gamma | 1080 | 3.95 | 0.13 |
| Cost of treatment, US$ * | Gamma | 1080 | 5.83 | 0.21 |
| * Average values in the sampled population. Probabilistic sensitivity analysis also accounted for differences in these values by age groups (<15 years, ≥15 years), place of residence (urban, rural areas), and type of healthcare facility (conventional, private). | | | | |

# **Table S9. Economic costs associated with uncomplicated malaria for all cases reported in 2017 in the DRC, according to multi-way probabilistic sensitivity analyses**

| **Costs** | **Estimate (US$)** | **Uncertainty limits**  **[95%CI]** |
| --- | --- | --- |
| Average economic cost | 36.3 | [35.5; 37.2] |
| Average direct cost | 16.7 | [16.2; 17.1] |
| Average indirect cost | 19.8 | [18.9; 20.3] |
| Total economic costs in DRC | 503,837,635.2 | [492,664,040.3; 515,094,408.3] |
| Direct costs in DRC | 231,359,957.5 | [225,814,749.1; 237,016,070.0] |
| Indirect costs in DRC | 274,376,911.6 | [263,258,768.8; 281,460,915.4] |
